# Neuroprotection Induced by Energy and Protein-Energy Undernutrition Is Phase-Dependent After Focal Cerebral Ischemia in Mice

**DOI:** 10.1007/s12975-019-00700-3

**Published:** 2019-03-18

**Authors:** Tayana Silva de Carvalho, Eduardo H. Sanchez-Mendoza, Luiza M. Nascentes Melo, Adriana R. Schultz Moreira, Maryam Sardari, Egor Dzyubenko, Christoph Kleinschnitz, Dirk M. Hermann

**Affiliations:** Department of Neurology, University Hospital Essen, Hufelandstr. 55, 45122 Essen, Germany

**Keywords:** Cerebral blood flow, Diet modification, Ischemic stroke, Malnutrition, Neuroinflammation, Neuroprotection

## Abstract

Malnutrition predisposes to poor stroke outcome. In animal models, undernutrition protected against ischemic injury in some, but not in other studies. In view of diverse stroke models and food restriction paradigms, the consequences of undernutrition are poorly understood. Herein, we exposed mice to energy-reduced and protein-energy-reduced diets for 7–30 days and subsequently induced intraluminal middle cerebral artery occlusion. Undernutrition phase dependently influenced ischemic injury. Short-lasting 7 days of protein-energy undernutrition, but not energy undernutrition, decreased post-ischemic brain leukocyte infiltration and microglial activation and reduced brain *Il-1β* mRNA, but did not protect against ischemic injury. Fourteen days of energy and protein-energy undernutrition, on the other hand, reduced ischemic injury despite absence of anti-inflammatory effects. Anti-oxidant genes (*Sod-1*, *Sod-2*, and *Cat* mRNAs) were regulated in the liver and, to a lesser extent, the ischemic brain, indicating an adapted, compensated stage. Conversely, 30 days of energy and protein-energy undernutrition caused progressive animal exhaustion associated with post-ischemic hypoperfusion, rise of metabolic markers (*Sirt-1* and *Glut-1* mRNAs, Sirt-1 protein) in the ischemic brain, and reregulation of pro- and anti-oxidant markers (now also *Nox-4* and *Gpx-3* mRNAs) in the liver. In the latter condition, no neuroprotection was noted. Our study suggests an adaptation of metabolic systems that provides neuroprotection in a circumscribed time window.

**Electronic supplementary material:**

The online version of this article (10.1007/s12975-019-00700-3) contains supplementary material, which is available to authorized users.

## Introduction

Malnutrition predisposes to death, stroke, and poor stroke outcome in humans. In a systematic analysis of 57 prospective studies involving 894,576 adults that were followed up over 13 years, a low body mass index (BMI; < 20 kg/m^2^) was associated with increased total and vascular mortality [1]. Malnutrition increases stroke risk in addition to established vascular risk factors; as shown in the U.S. Renal Data System registry, a cohort of 8920 patients with end-stage renal disease, in which three markers of malnutrition, that is, low weight (hazard ratio 1.09 (confidence interval 1.00–1.18) per 25% decrease), low serum albumin (1.43 (1.17–1.74) per g/dl decrease), and investigator judgment of undernourishment (1.27 (1.01–1.61)) independently predicted stroke risk [2]. In 104 acute stroke patients, protein-energy malnutrition increased cortisol stress responses, predisposed them to urinary or respiratory infections, and reduced neurological recovery after 1 month [3].

Undernourishment may have various causes, including disease-associated wasting, denutrition of various causes (including denutrition post-stroke), or intended fasting, which have very different consequences for stroke outcome. Considering the high prevalence of undernutrition in stroke patients, ranging from 6 to 62% depending on hospital environments [4], relatively little is known about how undernutrition affects ischemic brain injury. This is in contrast to the vast literature about the role of overnutrition and obesity in the ischemic brain (e.g., see [5, 6]). Surprisingly, some experimental studies showed that continuous or intermittent food restriction reduces post-ischemic neurological deficits [7–11], whereas other studies found exacerbated or unchanged neurological impairments [12–15]. Some studies showed that undernutrition reduced histological brain injury [7–9, 16, 17], whereas other studies observed no effect [10–13]. Some studies reported anti-inflammatory effects related to undernutrition, that is, reduced microglial activation [15] and decreased interleukin (IL)-1β, IL-6, or tumor necrosis factor-α (TNF-α) levels in the brain and blood [9, 16, 17]. Some studies described an upregulation of the NAD-dependent deacetylase sirtuin-1 (Sirt-1) that conferred neuroprotection [8, 16], an upregulation of anti-oxidant heme oxidase-1 and chaperones [7, 17], or an upregulation [17] or downregulation [9] of the growth factors brain-derived neurotrophic factor (BDNF), glial cell–derived neurotrophic factor (GDNF), and fibroblast growth factor-2 (FGF-2).

The above experimental studies strongly differed in their study designs. These differences relate to ischemia models, and the type, duration, and severity of diet modification, which in some cases went along with deficiency of essential micronutrients and vitamins. The diverse nature of these studies precludes more general conclusions about the effect of undernutrition on the ischemic brain. To elucidate how an undernutrition, which affects the amount of energy and protein delivered, but does not withhold essential micronutrients or vitamins, influences ischemic injury, we herein exposed mice to two protocols of diet modification, that is, energy undernutrition or protein-energy undernutrition over 7, 14, or 30 days, and subsequently induced focal cerebral ischemia by intraluminal middle cerebral artery occlusion (MCAO).

## Materials and Methods

### Legal Issues, Statistical Planning, and Randomization

Experiments were approved by local government authorities (Bezirksregierung Düsseldorf) in accordance with E.U. guidelines (Directive 2010/63/EU) for the care and use of laboratory animals. Sample size calculations determined that 12 animals per group were required for neurological examinations and histochemical studies, given that the effect size was 1.167, the alpha error was 5%, and the beta error (1 statistical power) was 20%. Experimenters were blinded by a third person not involved in the assessments randomizing the animals, weighing, and providing the food pellets.

### Food Modifications and Murinometrics

Adult male C57BL6/j mice (8 weeks, 26–30 g; Harlan-Netherlands, Rossdorf, Germany) were randomized to three diets: (a) normal nutrition (C1000; 3518 kcal/kg, 20% protein (i.e., casein); Altromin, Lage, Germany), (b) energy-reduced nutrition (C1012 mod.; 1313 kcal/kg, 20% protein (casein); Altromin), and (c) protein-energy-reduced nutrition (C1003 mod.; 1300 kcal/kg, 8% protein (casein); Altromin). Diets were offered ad libitum over 7, 14, or 30 days. Animals were then submitted to 30 min intraluminal MCAO. Throughout the study, animals were housed in single cages in a 12 h:12 h light/dark cycle. Food consumption and calorie intake were measured daily. Body (i.e., nose-anus) length was determined prior to diet modification. Body weight and BMI were determined weekly. Stool changes and behavioral abnormalities, namely, spontaneous motor hypoactivity, were checked daily.

### Experimental Procedures

Mice were anesthetized with 1.0–1.5% isoflurane (30% O_2_, remainder N_2_O). Rectal temperature was maintained between 36.5 and 37.0 °C using a feedback-controlled heating system. Cerebral laser Doppler flow (LDF) was recorded using a flexible probe (Perimed, Järfälla, Sweden) attached to the skull overlying the core of the middle cerebral artery territory. A midline neck incision was made. The left common and external carotid arteries were isolated and ligated, and the internal carotid artery was temporarily clipped. A silicon-coated nylon monofilament (0.21-mm tip diameter; Doccol, Sharon, MA, USA) was introduced through a small incision of the common carotid artery and advanced to the circle of Willis for MCAO [5, 6]. Reperfusion was initiated by monofilament removal after 30 min. Thirty minutes of MCAO was chosen, since this model induced reproducible injury of the striatum and the most lateral cortex with little animal dropouts. After surgery, wounds were carefully sutured and anesthesia was discontinued. Twenty-four hours later, animals were evaluated using the Clark score [18], which captures general and focal neurological deficits. Immediately before sacrifice, plasma samples were obtained by cardiac puncture after 5 h fasting that were used for analysis of total cholesterol, low-density lipoprotein cholesterol (LDL), triglycerides, and glucose levels (ADVIA® 2400; Siemens, Erlangen, Germany). One set of animals (*n* = 12/group) was transcardially perfused with normal saline followed by 4% paraformaldehyde. The animals’ brains were cut into 20-μm-thick coronal sections for histochemical studies. Another set of animals (*n* = 6/group) was transcardially perfused with normal saline. From the animals’ brains, tissue samples were collected from the middle cerebral artery territory for Western blots and real-time quantitative polymerase chain reaction (qPCR) studies. For this purpose, a 2-mm-thick coronal brain slice ranging from 1 mm rostral to 1 mm caudal to the bregma was prepared, from which a triangular slice containing the striatum and the most lateral parietal cortex was dissected. This selection strategy was chosen to exclude partial volume effects of infarct reductions or expansions on gene expression results. From the same animals, liver samples were also obtained.

### Infarct Volumetry

Coronal sections collected at millimeter intervals across the brain were stained with cresyl violet. Infarct volume was determined by subtracting the area of healthy tissue in the ischemic hemisphere from that in the contralesional hemisphere [5, 6].

### Immunohistochemistry of IgG Extravasation

Brain sections obtained from the rostrocaudal level of the midstriatum were rinsed for 20 min in 0.3% H_2_O_2_ in 70% methanol/0.1 M phosphate-buffered saline (PBS), immersed in 0.1 M PBS containing 5% bovine serum albumin (BSA) (05470; Sigma-Aldrich, Darmstadt, Germany), and incubated for 1 h in biotinylated anti-mouse IgG (1:100; Santa Cruz, Heidelberg, Germany), followed by diaminobenzidine (DAB) tetrahydrochloride (D5905; Sigma-Aldrich) staining with an avidin-biotin complex peroxidase kit (Vectastain Elite; Vector Labs, Burlingame, CA, USA) [5]. IgG extravasation was analyzed by measuring the area of IgG leakage.

### Terminal Deoxynucleotidyl Transferase–Mediated dUTP Nick End Labeling

Adjacent brain sections were subjected to terminal deoxynucleotidyl transferase–mediated dUTP nick end labeling (TUNEL) using a commercially available In Situ Cell Death Detection Kit (Roche, Mannheim, Germany) [5, 6]. TUNEL+, that is, DNA-fragmented cells were evaluated under a Zeiss AxioObserver.Z1 microscope equipped with Apotome optical sectioning by counting the total number of labeled cells in the striatum.

### Immunohistochemistry for Neuronal, Microglial, Astrocytic, and Inflammation Markers

Adjacent sections were immersed in 0.1 M PBS containing 0.3% Triton X-100 (PBS-T) and 5% normal donkey serum (D9663; Sigma-Aldrich). Sections were incubated overnight at 4 °C in monoclonal rabbit anti-NeuN (1:400; ab177487; Abcam, Cambridge, UK), monoclonal rat anti-CD45 (1:200; 550539; BD Biosciences, Heidelberg, Germany), polyclonal rabbit anti-ionized calcium binding adaptor protein (Iba)-1 (1:500; Wako Chemicals, Neuss, Germany), monoclonal rat anti-glial fibrillary acidic protein (GFAP) (1:200; 130300; Invitrogen, Dublin, Ireland), or polyclonal rabbit anti-inducible nitric oxide synthase (iNOS) (1:100; sc-650; Santa Cruz, CA, USA) antibodies that were detected with Alexa Fluor-488– or Alexa Fluor-594–labeled secondary antibodies (NeuN, Iba-1, GFAP, and iNOS) or biotinylated secondary antibodies followed by DAB staining with an avidin-biotin complex peroxidase kit (Vectastain Elite, Burlingame, CA, USA) (CD45). NeuN, Iba-1, GFAP, and iNOS labelings were counterstained with 4′,6-diamidino-2-phenylindole (DAPI) (D9542; Sigma-Aldrich). Sections were evaluated under a motorized Zeiss AxioObserver.Z1 inverted epifluorescence microscope equipped with Apotome optical sectioning (NeuN, Iba-1, GFAP, and iNOS) or an Olympus X52 microscope (CD45) by counting the total number of NeuN+, CD45+, or iNOS+ cells in the striatum, in which ischemic injury is most reproducible, or analyzing the area covered by activated microglia (Iba-1) or reactive astrocytes (GFAP). The latter analysis was preferred to cell counting, since individual cells could not always unequivocally be discriminated. The latter data were shown as percent changes.

### Real-Time Quantitative Polymerase Chain Reaction

From the brain and liver tissue samples, messenger RNA (mRNA) was extracted using an RNeasy Mini Kit (Qiagen, Hilden, Germany). mRNA was converted to cDNA using a high-capacity RNA-to-cDNA kit (Thermo Fisher Scientific, Langenselbold, Germany). Real-time qPCR was performed in a StepOnePlus real-time PCR system using primers selected by the PubMed primer-BLAST tool (https://blast.ncbi.nlm.nih.gov/) (Suppl. Table 1). The efficiency of these primers had been confirmed in melting curves. *β-Glucuronidase* (*β-Gluc*) was used as a housekeeping gene; the brain and liver tissue from healthy mice served as control. Results were quantified using the 2^−∆∆*Ct*^ method. PCR were performed in triplicate, of which mean values were formed for each mouse.

### Western Blots

During the mRNA extraction, protein samples were collected after bromochloropropane (B9673; Sigma-Aldrich) separation. Ethanol was added and samples centrifuged at 12.000*g* for 5 min. This procedure was repeated twice. The resulting pellet was suspended in 4% sodium dodecyl sulfate (SDS) (436143; Sigma-Aldrich). Protein content was measured using the Bradford method. Equal amounts of protein (20 μg) were loaded on 10% SDS-polyacrylamide gels, submitted to SDS-polyacrylamide gel electrophoresis (PAGE), and transferred onto polyvinylidene fluoride (PVDF) membranes (Bio-Rad, Hercules, CA). Membranes were blocked by 5% non-fat-dried milk (M7409; Sigma-Aldrich) in 50 mM Tris-buffered saline (TBS) containing 0.1% Tween (P9416; Sigma-Aldrich) for 1 h at room temperature, washed, and incubated overnight at 4 °C with monoclonal rabbit anti-Sirt-1 (1:2000; ab32441; Abcam) and polyclonal rabbit anti-β-actin (1:10000; 4967; Cell Signaling, Frankfurt, Germany) antibodies. The next day, membranes were washed and incubated with secondary donkey anti-rabbit antibody. Blots were revealed using a chemiluminescence kit and scanned using a myECL Imager (Thermo Fisher Scientific). Sirt-1 abundance was densitometrically evaluated in three independent experiments. The relative abundance of Sirt-1 was normalized to protein loading as determined in β-actin blots.

### Statistics

Statistical analyses were performed using SPSS for Windows. Nutritional data and LDF recordings were analyzed by two-way repeated measurement ANOVA followed by unpaired *t* tests as post hoc tests. Neurological deficits and histochemical data were analyzed by one-way ANOVA followed by Tukey post hoc tests (for normally distributed results) or Kruskal-Wallis tests (for non-normally distributed results). Real-time qPCR data were compared by pairwise *t* tests. To explore the relationship of calorie intake with infarct volume and neurological deficits, two-tailed Pearson’s correlations were computed. Nutritional data, LDF recordings, and real-time qPCR data are presented as mean ± S.D. values. Neurological deficits, histochemical data, and Western blots are shown as median ± interquartile range box plots with minimum/maximum data as whiskers. *p* values < 0.05 were defined to indicate statistical significance.

## Results

### Food Modification Induces Weight Loss and Malabsorption Syndrome

Murinometric analyses revealed progressive body weight (Suppl. Fig. 1A–C) and body mass index (BMI) (Suppl. Fig. 1D–F) loss (each by ~ 20%) over up to 30 days in mice exposed to energy and protein-energy undernutrition. Although the total amount of food ingested was elevated in mice on modified diets (Suppl. Fig. 1G–I), calorie intake was consistently reduced at all time points at which ischemia was subsequently induced (Suppl. Fig. 1J–L). Upon diet modification, stool samples progressively increased in size and adopted a pale color (Suppl. Fig. 2; Suppl. Table 2), indicative of malabsorption syndrome. With progressive undernutrition, thin blood beddings were sometimes found on stool samples (Suppl. Table 2). Motor hypoactivity was frequently noted shortly before MCAO (Suppl. Table 2).

Plasma LDL, which constitutes a comparably small percentage of total cholesterol in mice, since mice lack cholesterol ester transfer protein (CETP) [19], was decreased in mice exposed to protein-energy undernutrition for 7 days, but not for 14 or 30 days (Suppl. Table 3). Total cholesterol, triglycerides, and glucose were not influenced by energy or protein-energy undernutrition (Suppl. Table 3).

### Energy and Protein-Energy Undernutrition Attenuate Ischemic Injury in a Limited Time Window

**Fig. 1:**
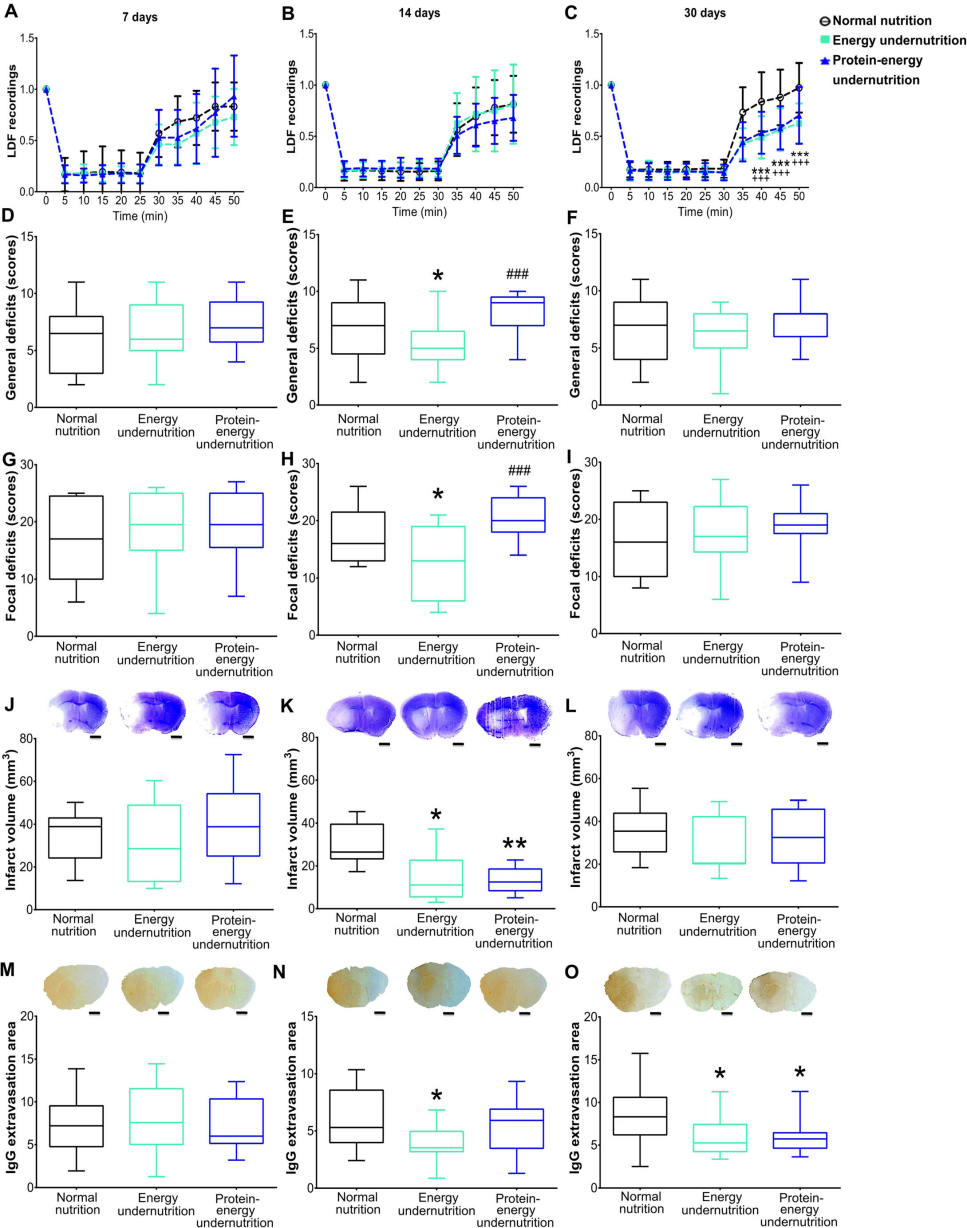
Energy and protein-energy undernutrition reduce ischemic injury in a defined time window. (A–C) Laser Doppler flow (LDF) recordings above the core of the middle cerebral artery territory, (D–F) general neurological deficits evaluated by the Clark score, (G–I) focal neurological deficits examined by the Clark score, (J–L) infarct volume outlined on cresyl violet-stained brain sections, and (M–O) blood-brain barrier permeability in the striatum assessed by IgG extravasation analysis in mice exposed to normal nutrition, energy undernutrition, or protein-energy undernutrition for 7 days (A, D, G, J, M), 14 days (B, E, H, K, N), or 30 days (C, F, I, L, O), followed by 30 min intraluminal MCAO and 24 h reperfusion. Representative cresyl violet stainings and IgG immunostainings are shown. Bars (in (J–O)), 1 mm. ***p < 0.001 for energy undernutrition compared with normal nutrition/^+++^*p* < 0.001 for protein-energy undernutrition compared with normal nutrition (in (A-C); *n* = 12 animals/group). *p < 0.05/**p < 0.01 compared with normal nutrition/^###^*p* < 0.001 compared with energy undernutrition (in (D–O); *n* = 12 animals/group)

In mice receiving non-modified diet, cerebral LDF decreased to ~ 15–20% of baseline values during MCAO, followed by the restoration of LDF to baseline values within 20 min after monofilament removal (Fig. 1A–C). LDF patterns did not differ in mice exposed to energy or protein-energy undernutrition for 7 or 14 days (Fig. 1A, B), whereas decreased reperfusion indicative of hemodynamic impairment was noted in mice exposed to prolonged energy and protein-energy undernutrition for 30 days (Fig. 1C). Neurological examination 24 h after MCAO revealed that energy undernutrition but not protein-energy undernutrition for 14 days decreased general and focal neurological deficits (Fig. 1E, H), whereas diet modification for 7 or 30 days did not influence neurological performance (Fig. 1D, F, G, I). Both energy and protein-energy undernutrition significantly reduced infarct volume, when imposed for 14 days, but not 7 or 30 days (Fig. 1J–L). IgG extravasation, a measure of disturbed blood-brain barrier integrity, was reduced by energy undernutrition for 14 or 30 days and protein-energy undernutrition for 30 days (Fig. 1M–O).

Our data were indicative of three stages of undernutrition: an initial adaptation stage (that is, 7 days after diet modification), in which ischemic injury is not influenced by energy and protein-energy undernutrition, which is followed by a compensated stage (that is, 14 days after diet modification), in which brain tissue is protected against ischemia, and an exhausted stage (that is, after 30 days), in which neuroprotection is lost as a consequence of post-ischemic hemodynamic impairments. Pearson’s correlations revealed that calorie intake in the adaptation stage was negatively correlated with general neurological deficits (*r* = − 0.275, *p* = 0.02), but not with infarct volume or focal deficits (Suppl. Fig. 3). In the compensated stage, calorie intake was positively correlated with infarct volume (*r* = 0.572, *p* < 0.001), but not with neurological deficits (Suppl. Fig. 3). In the exhausted stage, no correlations of calorie intake with infarct volume or neurological deficits were found (Suppl. Fig. 3).

### Undernutrition Differentially Influences Neuronal Survival, Brain Leukocyte Infiltration, and Microglial Activation

**Fig. 2:**
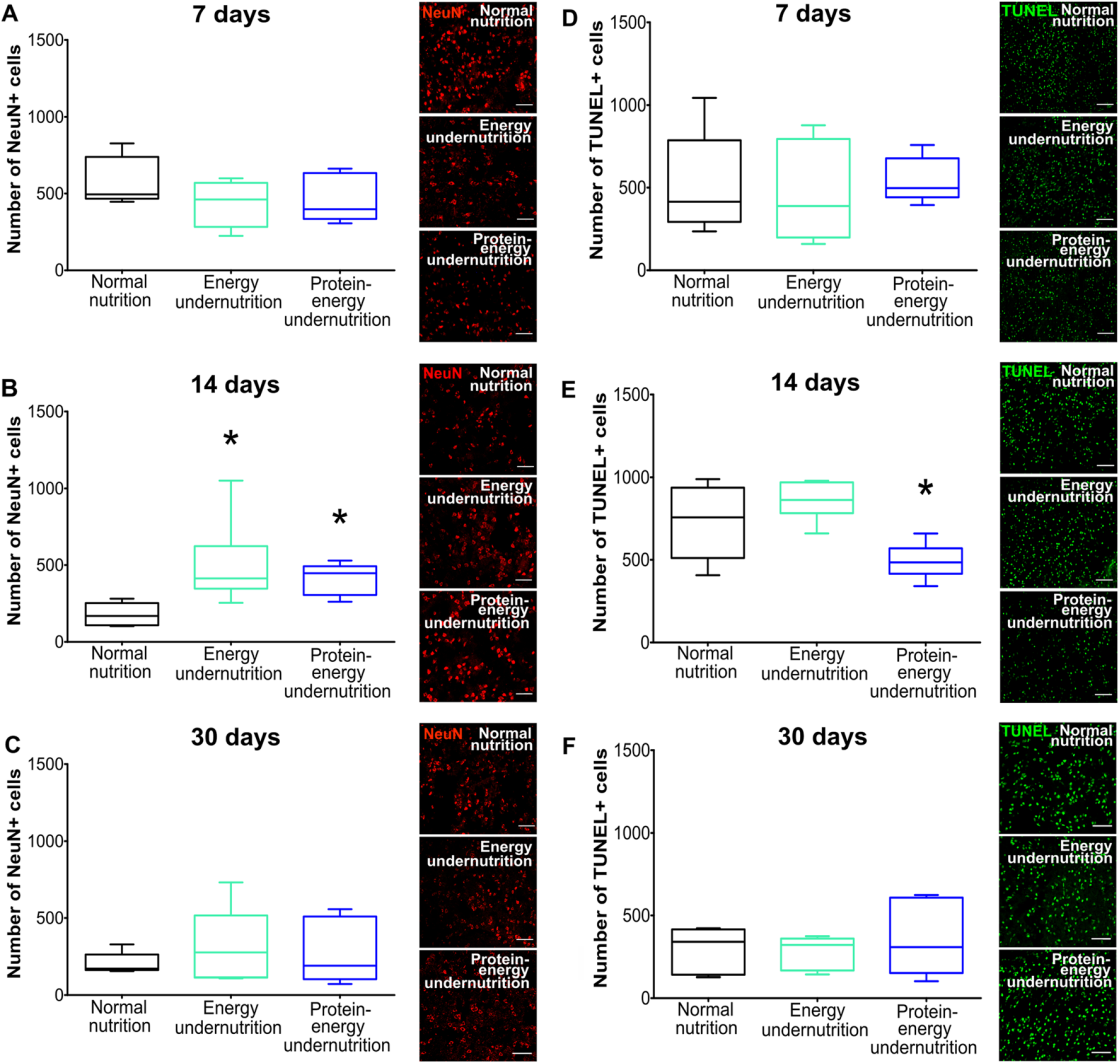
Undernutrition increases neuronal survival in a limited time window. (A–C) Number of NeuN+ surviving neurons and (D–F) number of DNA-fragmented, that is, irreversibly injured TUNEL+ cells in the ischemic striatum of mice exposed to normal nutrition, energy undernutrition, or protein-energy undernutrition for 7 days (A, D), 14 days (B, E), or 30 days (C, F), followed by 30 min intraluminal MCAO and 24 h reperfusion. Representative microphotographs are shown. Bars, 100 μm. *p < 0.05 compared with normal nutrition (*n* = 12 animals/group)

**Fig. 3:**
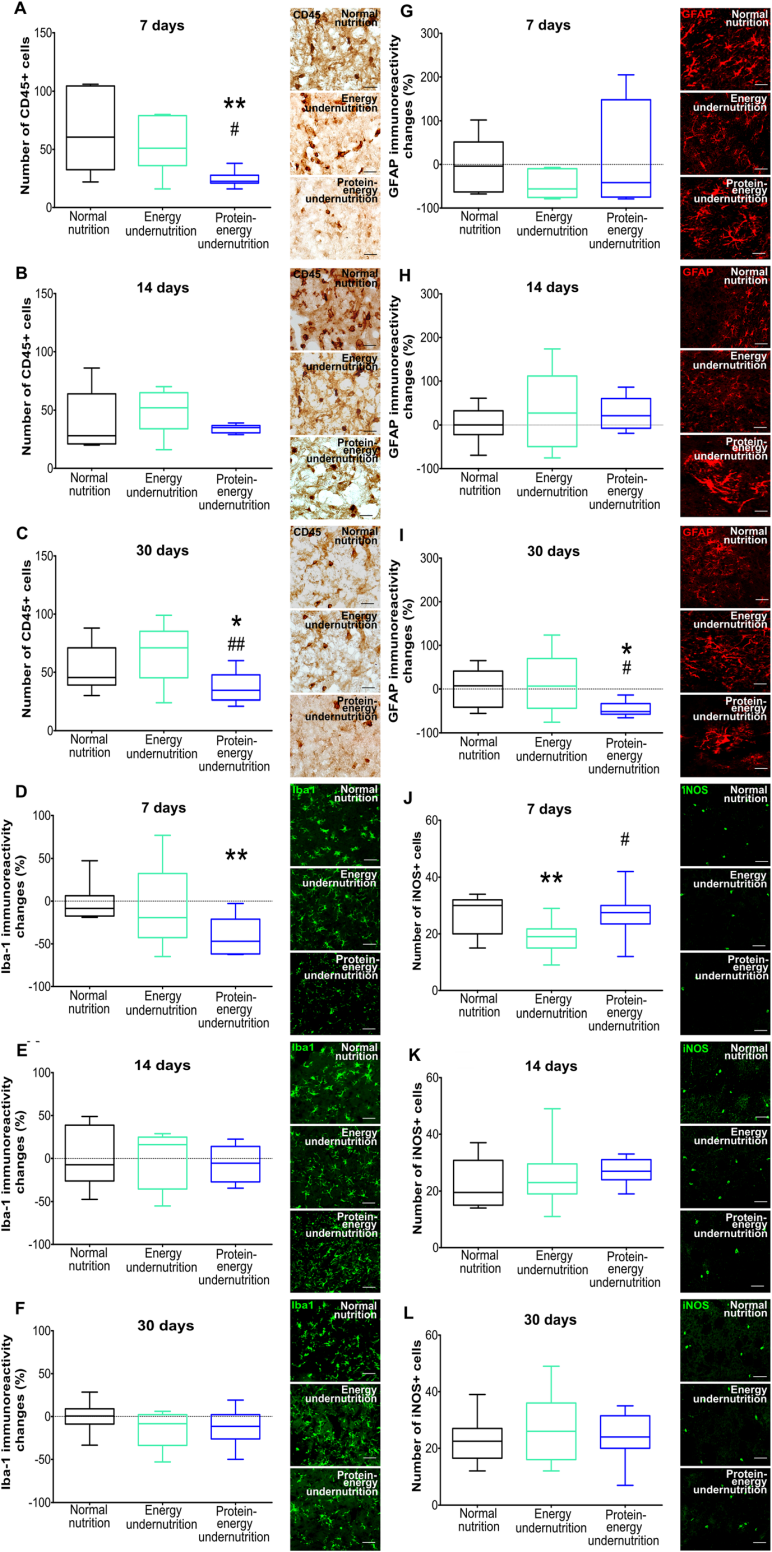
Undernutrition differentially influences brain leukocyte infiltration, microglial activation, and astroglial reactivity. (A–C) Number of CD45+ leukocytes, (D–F) immunoreactivity for microglia marker Iba-1, (G–I) immunoreactivity for astrocytic marker GFAP, and (J–L) number of iNOS+ cells in the ischemic striatum of mice exposed to normal nutrition, energy undernutrition, or protein-energy undernutrition for 7 days (A, D, G, J), 14 days (B, E, H, K), or 30 days (C, F, I, L), followed by 30 min intraluminal MCAO and 24 h reperfusion. Representative microphotographs are shown. Bars, 100 μm. *p < 0.05/**p < 0.01 compared with normal nutrition/^#^p < 0.05/^##^*p* < 0.01 compared with energy undernutrition (*n* = 12 animals/group)

Immunohistochemical studies showed that energy and protein-energy undernutrition for 14 days increased the density of surviving NeuN+ neurons in the ischemic striatum (Fig. 2B) and that protein-energy undernutrition reduced the density of DNA-fragmented, that is, irreversibly injured TUNEL+ cells (Fig. 2E). Undernutrition did not influence neuronal survival (Fig. 2A, C) or cell injury (Fig. 2D, F), when imposed for 7 or 30 days. Protein-energy undernutrition decreased brain leukocyte infiltration, as assessed by CD45 immunohistochemistry (Fig. 3A), and microglial activation, as evaluated by Iba-1 immunohistochemistry (Fig. 3D), when imposed for 7 days. Energy undernutrition for 7 days reduced the density of iNOS+ cells (Fig. 3J), which had the size and shape of microglia. Interestingly, diet modification for 14 days did not alter brain leukocyte infiltration, microglial activation, or iNOS formation (Fig. 3B, E, K). Protein-energy undernutrition for 30 days reduced the brain infiltration of CD45+ leukocytes (Fig. 3C) and decreased astrocytic GFAP immunoreactivity (Fig. 3I).

### Undernutrition Regulates Metabolism-Related, Inflammatory, and Anti-Oxidant Genes in Ischemic Brain Tissue

**Fig. 4:**
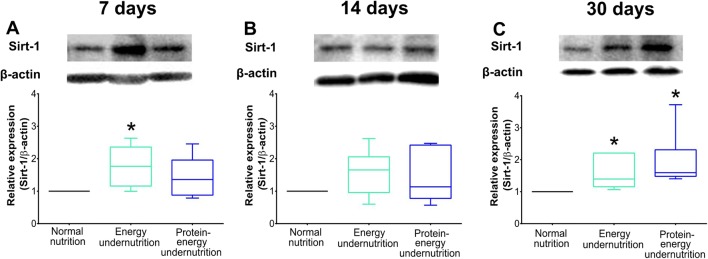
Undernutrition regulates Sirt-1 protein in the ischemic brain. Western blot analysis of Sirt-1 protein in ischemic brain tissue of mice exposed to normal nutrition, energy undernutrition, or protein-energy undernutrition for (A) 7 days, (B) 14 days, or (C) 30 days, followed by 30 min intraluminal MCAO and 24 h reperfusion. Representative Western blots are also shown. *p < 0.05 compared with normal nutrition (*n* = 6 animals/group)

Real-time qPCR showed that undernutrition regulated metabolism-related, inflammatory, and anti-oxidant genes in the ischemic brain. *Sirtuin-1* (*Sirt-1*) mRNA, which encodes for an NAD-dependent deacetylase that stabilizes mitochondrial function and metabolism partly by deacetylating the transcription regulator peroxisome proliferator-activated receptor-γ coactivator-1α (PGC-1α) [20], and *glucose transporter-1* (*Glut-1*) mRNA, which encodes for a membrane transporter promoting glucose uptake in brain cells [21], were upregulated in the ischemic brain of mice exposed to 7 days energy undernutrition (Table 1), as was Sirt-1 protein, as shown in the Western blots (Fig. 4A). *Il-1β* mRNA was downregulated by 7 days protein-energy undernutrition (Table 1). Only subtle gene expression changes were noted in the ischemic brain of mice exposed to 14 days undernutrition, that is, an elevation of *superoxide dismutase* (*Sod*)*-1* mRNA, which encodes for a dismutase degrading superoxide anions, in mice exposed to protein-energy-reduced diet (Table 1). *Sirt-1* and *Glut-1* mRNAs (Table 1) as well as Sirt-1 protein (Fig. 4B) did not differ from mice receiving non-modified diet, indicating that the metabolic needs of the tissue were adapted to the reduced energy supply. In the ischemic brain of mice exposed to prolonged energy or protein-energy undernutrition for 30 days, *Sirt-1* mRNA (Table 1) and Sirt-1 protein (Fig. 4C) were increased, and in the ischemic brain of mice exposed to prolonged protein-energy undernutrition for 30 days, *Glut-1* and *Sod-1* mRNA (Table 1) were elevated. Conversely, *insulin-like growth factor-1* (*Igf-1*) mRNA, which encodes for a growth factor with insulin-like properties, *Il-1β* mRNA, and *nuclear factor*-*κb* (*Nf*-*κb*) mRNA, which encodes for a transcription factor, were reduced by energy or protein-energy undernutrition (Table 1). The responses of *Sirt-1*, *Glut-1*, and *Igf-1* were interpreted as effort to maintain brain tissue glucose supply and confine metabolic needs in face of energy reserves which faded.

**Table 1:** Undernutrition regulates metabolism-related, inflammatory, and anti-oxidant genes in the ischemic brain

Brain tissue	*Sirt-1*	*Igf-1*	*Insr*	*Glut-1*	*Il-1β*	*Nf-κb*	*Sod-1*	*Gpx-3*
7 days								
Normal nutrition	1.13 ± 0.60	3.60 ± 3.26	1.44 ± 0.61	1.18 ± 0.44	6.14 ± 3.26	1.40 ± 0.65	0.89 ± 0.34	0.13 ± 0.10
Energy undernutrition	1.87 ± 0.91*	3.94 ± 2.88	1.87 ± 0.86	1.99 ± 0.74*	3.68 ± 2.77	1.87 ± 0.69	1.38 ± 0.61	0.18 ± 0.09
Protein-energy undernutrition	1.12 ± 0.30	2.44 ± 1.83	1.37 ± 0.49	1.65 ± 0.50	1.71 ± 0.37*	1.30 ± 0.36^**#**^	1.15 ± 0.54	0.12 ± 0.03
14 days								
Normal nutrition	0.60 ± 0.23	1.15 ± 0.58	1.33 ± 1.07	1.17 ± 0.44	3.50 ± 1.59	2.91 ± 1.23	0.90 ± 0.27	0.28 ± 0.15
Energy undernutrition	0.51 ± 0.21	1.86 ± 0.83	0.97 ± 0.33	1.11 ± 0.55	3.16 ± 1.62	2.32 ± 1.10	0.90 ± 0.32	0.19 ± 0.05
Protein-energy undernutrition	0.70 ± 0.36	2.01 ± 2.88	1.15 ± 1.11	0.87 ± 0.47	3.00 ± 2.69	3.30 ± 2.42	1.76 ± 0.86*	0.26 ± 0.14
30 days								
Normal nutrition	0.28 ± 0.11	1.84 ± 1.12	0.69 ± 0.28	1.09 ± 0.56	5.72 ± 3.18	2.75 ± 1.34	0.57 ± 0.12	0.48 ± 0.50
Energy undernutrition	0.63 ± 0.27*	0.77 ± 0.37*	0.74 ± 0.17	1.72 ± 0.75	1.11 ± 0.45**	1.44 ± 0.42*	0.71 ± 0.24	0.45 ± 0.37
Protein-energy undernutrition	0.66 ± 0.25*	2.11 ± 0.80^**#**^	0.98 ± 0.61	2.02 ± 0.76*	1.50 ± 0.68**	1.39 ± 0.35*	0.99 ± 0.39*^#^	0.63 ± 0.45

*p < 0.05/**p < 0.01 compared with corresponding normal nutrition; ^#^*p* < 0.05 compared with corresponding energy undernutrition (*n* = 6 animals/group, analyzed in triplicate)

### Undernutrition Regulates Metabolism-Related, Pro-oxidant, and Anti-oxidant Genes in the Liver

Real-time qPCR showed that protein-energy undernutrition over 7 days downregulated *catalase* (*Cat*) mRNA, which encodes for a protein that degrades peroxides formed by dismutases, in the liver (Table 2). After 14 days protein-energy undernutrition, *Sod-1* mRNA was increased, whereas *glucose transporter-2* (*Glut-2*) mRNA, *Sod-2* mRNA, which encodes for another dismutase, and *Cat* mRNA were reduced, as was *Sod-2* mRNA after 14 days energy undernutrition (Table 2). Prolonged energy and protein-energy undernutrition over 30 days downregulated *NADPH oxidase-4* (*Nox-4*) mRNA, which encodes for a protein that catalyzes the production of superoxide free radical by transferring one electron to oxygen from NADP, and anti-oxidant *Sod-2* mRNA (Table 2). Prolonged energy undernutrition over 30 days upregulated *glutathione peroxidase-3* (*Gpx-3*) mRNA (Table 2), which encodes for another peroxidase.

**Table 2:** Undernutrition regulates metabolism-related, pro-oxidant, and anti-oxidant genes in the liver

Liver tissue	*Sirt-1*	*Igf-1*	*Insr*	*Glut-2*	*Nf-κb*	*Nox-4*	*Sod-1*	*Sod-2*	*Gpx-3*	*Cat*
7 days										
Normal nutrition	0.94 ± 0.45	0.75 ± 0.43	0.40 ± 0.48	0.53 ± 0.64	1.66 ± 0.87	1.15 ± 0.83	0.74 ± 0.26	1.07 ± 0.42	1.40 ± 1.29	0.54 ± 0.37
Energy undernutrition	1.31 ± 0.70	0.80 ± 0.63	0.33 ± 0.41	1.03 ± 0.76	1.57 ± 0.80	1.34 ± 1.02	1.04 ± 0.35	1.72 ± 0.98	1.70 ± 0.73	0.38 ± 0.34
Protein-energy undernutrition	1.09 ± 0.43	0.77 ± 0.70	0.26 ± 0.45	0.88 ± 0.78	1.45 ± 1.05	0.97 ± 0.88	0.80 ± 0.31	1.76 ± 1.04	2.72 ± 2.35	0.20 ± 0.20*
14 days										
Normal nutrition	0.45 ± 0.45	0.59 ± 0.37	0.50 ± 0.68	1.07 ± 0.26	1.05 ± 1.13	0.72 ± 0.51	0.97 ± 0.48	0.99 ± 0.22	4.23 ± 3.15	0.43 ± 0.47
Energy undernutrition	0.25 ± 0.15	1.01 ± 0.81	0.69 ± 0.71	0.68 ± 0.65	0.75 ± 0.43	0.62 ± 0.37	0.91 ± 0.21	0.59 ± 0.15*	3.72 ± 2.10	0.40 ± 0.36
Protein-energy undernutrition	0.27 ± 0.23	0.75 ± 0.47	0.23 ± 0.37	0.30 ± 0.33*	1.18 ± 1.36	0.86 ± 0.81	2.04 ± 0.72*^##^	0.46 ± 0.17**	3.48 ± 3.91	0.02 ± 0.02*
30 days										
Normal nutrition	0.74 ± 0.24	0.48 ± 0.47	0.24 ± 0.26	0.98 ± 0.65	0.67 ± 0.28	1.25 ± 0.36	1.04 ± 0.09	2.50 ± 1.86	0.84 ± 0.53	0.69 ± 0.54
Energy undernutrition	0.68 ± 0.39	0.43 ± 0.09	0.22 ± 0.19	0.56 ± 0.30	0.60 ± 0.36	0.61 ± 0.26**	1.13 ± 0.92	0.57 ± 0.07*	2.31 ± 0.76*	0.67 ± 0.47
Protein-energy undernutrition	1.07 ± 0.93	0.70 ± 0.78	0.29 ± 0.25	0.70 ± 0.58	1.20 ± 1.28	0.42 ± 0.11***	1.10 ± 0.84	0.62 ± 0.19*	2.60 ± 2.47	0.46 ± 0.27

*p < 0.05/**p < 0.01/***p < 0.001 compared with corresponding normal nutrition; ^##^*p* < 0.01 compared with corresponding energy undernutrition (*n* = 6 animals/group, analyzed in triplicate)

## Discussion

By exposing mice to energy-reduced or protein-energy-reduced diets for 7, 14, or 30 days, which were subsequently submitted to intraluminal MCAO, we show that energy and protein-energy undernutrition influences ischemic injury in a phase-dependent way. When exposed to modified diets for 7 days, combined protein-energy undernutrition reduced leukocyte infiltration and microglial activation and decreased the pro-inflammatory cytokine *Il-1β* mRNA in ischemic brain tissue. The brain was not protected against ischemia. Conversely, 14 days of energy or protein-energy undernutrition reduced ischemic injury and in the case of energy undernutrition decreased neurological deficits. Anti-inflammatory effects were absent in the ischemic brain. Genes encoding anti-oxidant enzymes (*Sod-1*, *Sod-2*, and *Cat* mRNA) were profoundly regulated in the liver and, to lesser extent, the brain. With prolonged energy or protein-energy undernutrition for 30 days, an exhausted stage evolved characterized by disturbed post-ischemic reperfusion, rise of metabolism markers in the brain (*Sirt-1* and *Glut-1* mRNA, Sirt-1 protein), downregulation of inflammatory markers in the brain (*Il-1β* and *Nf-κb* mRNA), and reregulation of pro-oxidant and anti-oxidant markers in the liver (now including *Nox-4* and *Gpx-3* mRNA), in which the brain was not protected against ischemia.

Previous studies in animal models of focal cerebral ischemia found that undernutrition reduces brain injury [7–9, 16, 17], whereas other studies in models of focal or global ischemia showed that ischemic injury was unchanged by undernutrition [10–13]. A variety of studies in models of focal or global ischemia observed a significant reduction of stroke-induced neurological deficits [7–11], whereas other studies, again in focal or global ischemia, noted exacerbated or unchanged deficits [12–15]. Major differences in these studies relate to the type of undernutrition. As such, undernutrition was induced by (a) reducing food access to 60 or 70% of the average amount of control animals for 4 weeks to 3 months [8, 10, 13, 16], (b) reducing food protein content to 0 to 12% for 6 days to 4 weeks [9, 12, 14, 15], or (c) intermittent fasting on alternate days or twice per week for one to several months [7, 11, 17]. While reducing the amount of food access similarly reduces protein and energy consumption, the reduction of protein content to 0–2% results in a reduction in the total amount of food ingested, since the animals refuse this chow [9, 12, 14, 15]. In such animals, combined protein-energy malnutrition is noted. In order to prevent exhaustion of the animals’ energy state, we decided to use a strategy, in which we applied modified diets ad libitum to mice. Essential minerals, micronutrients, and vitamins were adequately complemented in these diets. The use of an energy-reduced and protein-energy-reduced diet allowed us to discriminate both types of undernutrition.

Previous studies reported that neuroprotection in response to food restriction or undernutrition involves anti-inflammatory effects, that is, inhibition of microglial activation [15], downregulation of *Il-1β*, *Il-6*, *Tnf-α*, *Cxc-motif ligand-1* (*Cxcl-1*), and *intercellular adhesion molecule-1* (*Icam-1*) mRNA [9] or downregulation of IL-1β, IL-6 and TNF-α protein [16, 17] in the brain and the blood. Interestingly, in our study, diet modification reduced leukocyte infiltration, microglial activation, and *Il-1β* and *Nf-κb* mRNA levels in the ischemic brain under conditions not associated with neuroprotection, i.e., when modified chows were imposed for 7 or 30 days. After 14 days energy or protein-energy undernutrition, when neuroprotective effects were noted, anti-inflammatory effects were absent in the brain. Thus, diet-induced neuroprotection dissociated from anti-inflammation. That food deprivation reduces the brain infiltration of leukocytes has to the best of our knowledge not been shown.

Earlier studies found that undernutrition increases brain levels of the NAD-dependent deacetylase Sirt-1 [8, 16]. Sirt-1 has a large variety of actions in the healthy and injured brains, stabilizing mitochondrial function and metabolism in response to energy deprivation partly by deacetylating the transcription regulator PGC-1α [20]. Via multiple downstream targets, mitochondrial energy coupling is promoted, reactive oxygen species formation reduced, anti-oxidant capacity increased, and DNA repair enhanced [20]. In our study, *Sirt-1* mRNA and *Sirt-1* protein were elevated when energy or protein-energy undernutrition had been imposed for 7 or 30 days. Under these conditions, no neuroprotection was noted. Interestingly, Sirt-1^*−/−*^ was previously found to exacerbate ischemic injury in mice exposed to intraluminal MCAO but failed to abolish protective effects of energy restriction [16]. The combined evidence of these data suggests that Sirt-1 acts as a regulator of metabolism-related, pro-oxidant, and anti-oxidant genes, but does not contribute to diet-induced neuroprotection. In fact, *Sirt-1* mRNA and Sirt-1 protein elevation in our study were associated with the regulation of a broad set of downstream mRNAs, namely *Igf-1*, *Glut-1*, *Il-1β*, *Nf-κb*, *Sod-1*, and *Gpx*. Upregulation of anti-oxidant heme oxidase-1 has previously been reported in ischemic brains of mice exposed to alternate fasting or protein-energy undernutrition [7, 17]. The regulation of *Igf-1*, *Glut-1*, *Nf-κb*, *Sod-1*, and *Gpx* mRNAs in ischemic brains of animals exposed to energy or protein-energy undernutrition is new.

In our study, surprisingly modest metabolic, anti-inflammatory, and anti-oxidant changes were noted in animals exposed to 14 days energy or protein-energy undernutrition, which exhibited neuroprotective effects. In the brain, metabolic, anti-inflammatory, and anti-oxidant changes were lacking. It should be noted that histochemical and gene expression changes were determined at the same time point, at which brain injury was assessed, i.e., at 24 h post-MCAO. Our data cannot exclude earlier histochemical or gene expression changes that had already disappeared by then. At 24 h post-MCAO, neither brain leukocyte infiltration nor microglial activation, which are mediators of secondary brain injury [22], were altered in response to energy or protein-energy undernutrition. Our data suggest that the previously reported neuroprotection in models of energy and protein-energy undernutrition may represent a state of ischemic tolerance rather than a true neuroprotective state. The preceding regulation of metabolism-related genes (*Sirt-1*, *Glut-1*) indicates that tissue energy demands had been adjusted which enabled the tissue to survive ischemic injury. Ischemic tolerance can similarly be induced by intermittent fasting in young and aged rats [7, 11, 17], suggesting that this type of endogenous protection might also be induced in elderly humans by repeated short-lasting food restriction episodes. Diet modification induces a series of physiological and biochemical responses that we did not examine in this study, such as changes in blood gases, as well as changes of plasma glucose and lipids that we evaluated prior to animal sacrifice but not at baseline. It should be noted that plasma glucose and lipid levels are influenced by ischemic stroke and anesthesia. All these factors were adequately controlled for in animals on normal diet.

Notably, the diet-induced neuroprotection vanished with progressive exhaustion of the animals’ nutrition state, that is, after 30 days of energy or protein-energy undernutrition, when post-ischemic hypoperfusion prevented survival-promoting effects. As such, in advanced undernourishment, observations in animals do not contradict clinical experience in human patients that malnutrition impairs stroke outcome. With this respect, malnutrition apparently resembles its opposite state, that is, overnutrition and obesity, for which it has been assumed for many years that it enhances ischemic stroke outcome [23]. This so-called obesity paradox has meanwhile been refuted [24]. In view of its clinical relevance, future studies should more stringently examine consequences of nutrition modifications for ischemic stroke and stroke recovery.

## Electronic supplementary material

ESM 1 (PDF 890 kb)
