# Supplementary material for: Neuroprotection Induced by Energy and Protein-Energy Undernutrition Is Phase-Dependent After Focal Cerebral Ischemia in Mice
Source: Transl Stroke Res. 2019 Mar 18;11(1):135–46. doi: 10.1007/s12975-019-00700-3 (PMC6957545; doi:10.1007/s12975-019-00700-3)
Supplement: Supplementary file 1 — (PDF 890 kb) [file 12975_2019_700_MOESM1_ESM.pdf]

Neuroprotection induced by energy and protein-energy undernutrition is phase-dependent after focal cerebral ischemia in mice

Tayana Silva de Carvalho, MSc; Eduardo H. Sanchez-Mendoza, PhD; Luiza M. Nascentes Melo, MSc; Adriana R. Schultz Moreira, PhD; Maryam Sardari, PhD; Egor Dzyubenko, PhD; Christoph Kleinschnitz, MD; Dirk M. Hermann, MD

Department of Neurology, University Hospital Essen, Essen, Germany

Running title: Neuroprotection by energy and protein-energy undernutrition

Translational Stroke Research

Correspondence:

Prof. Dirk M. Hermann, MD

Department of Neurology

University Hospital Essen

Hufelandstr. 55

D-45122 Essen, Germany

Phone: +49-201-723-2814, Fax: +49-201-723-5534

E-mail: dirk.hermann@uk-essen.de

Supplemental Figures:

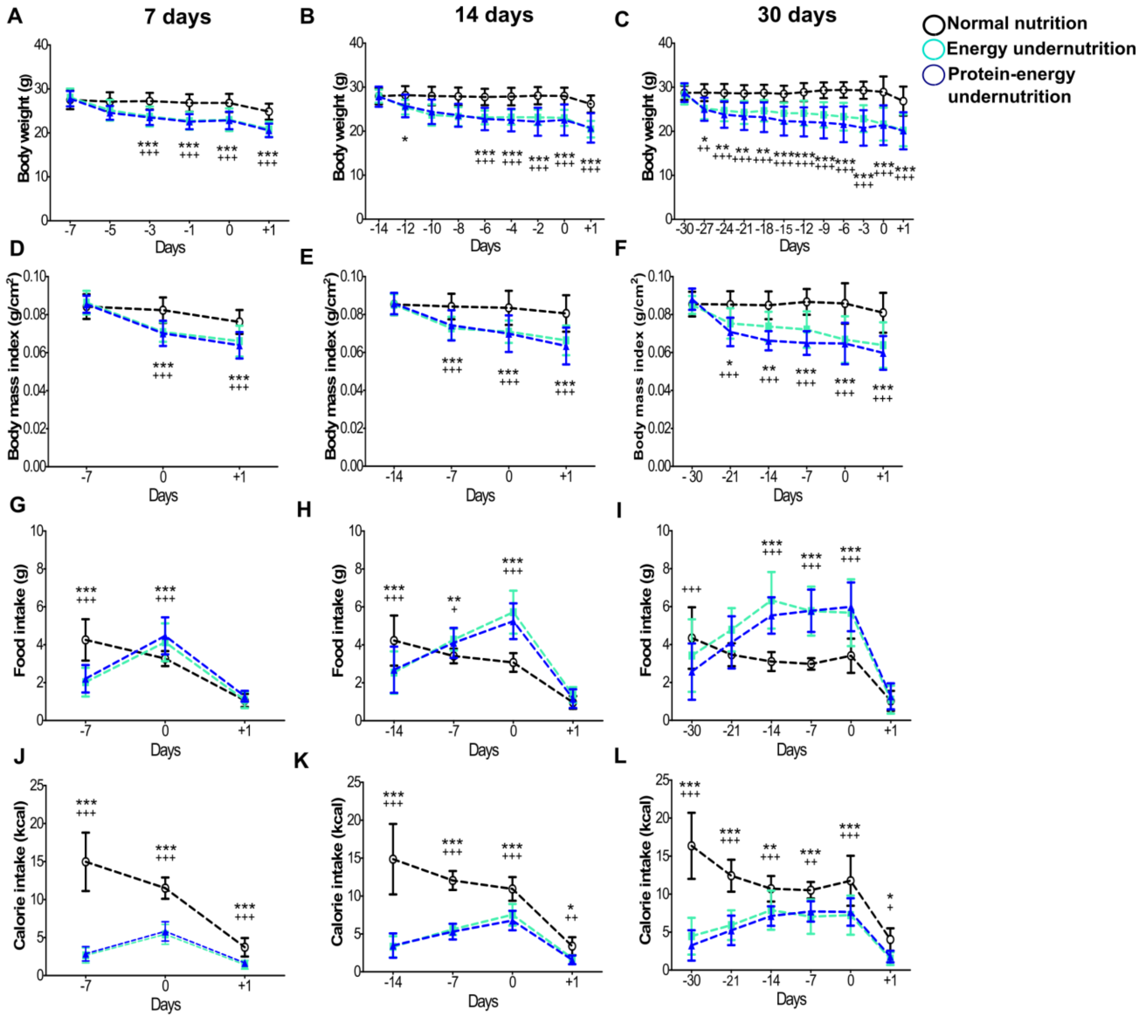

Supplemental Figure 1. Energy and protein-energy undernutrition induce weight loss despite increased food intake. (A-C) Body weight, **(D-F)** body-mass index (BMI), **(G-I)** daily food intake, **(J-L)** daily calorie intake in mice receiving normal nutrition, energy-reduced nutrition or protein-energy-reduced nutrition for 7 days **(A, D, G, J)**, 14 days **(B, E, H, K)**or 30 days **(C, F, I, L)**, followed by 30 minutes intraluminal MCAO and 24 hours reperfusion. The x-axis reflects the time-point with respect to tMCAO (=day 0). ***$p<0.001$ for energy undernutrition compared with normal nutrition/ $^{+++}p<0.001$ for protein-energy undernutrition compared with normal nutrition (n=12 animals/ group).

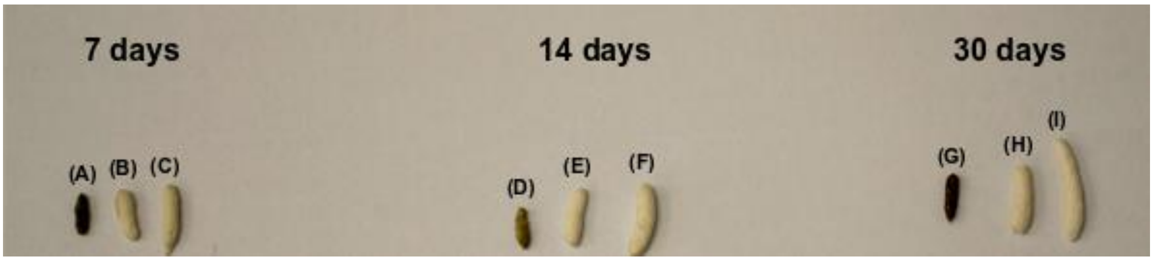

Supplemental Figure 2. Energy and protein-energy undernutrition induce stool changes indicative of malabsorption syndrome. Representative stool samples in animals exposed to **(A, D, G)** normal nutrition, **(B, E, H)** energy **under**nutrition and **(C, F, I)** protein-energy **under**nutrition over 7 days **(A-C)**, 14 days **(D-F)** or 30 days **(G-I)**. Stool samples were collected on the last day prior to MCAO.

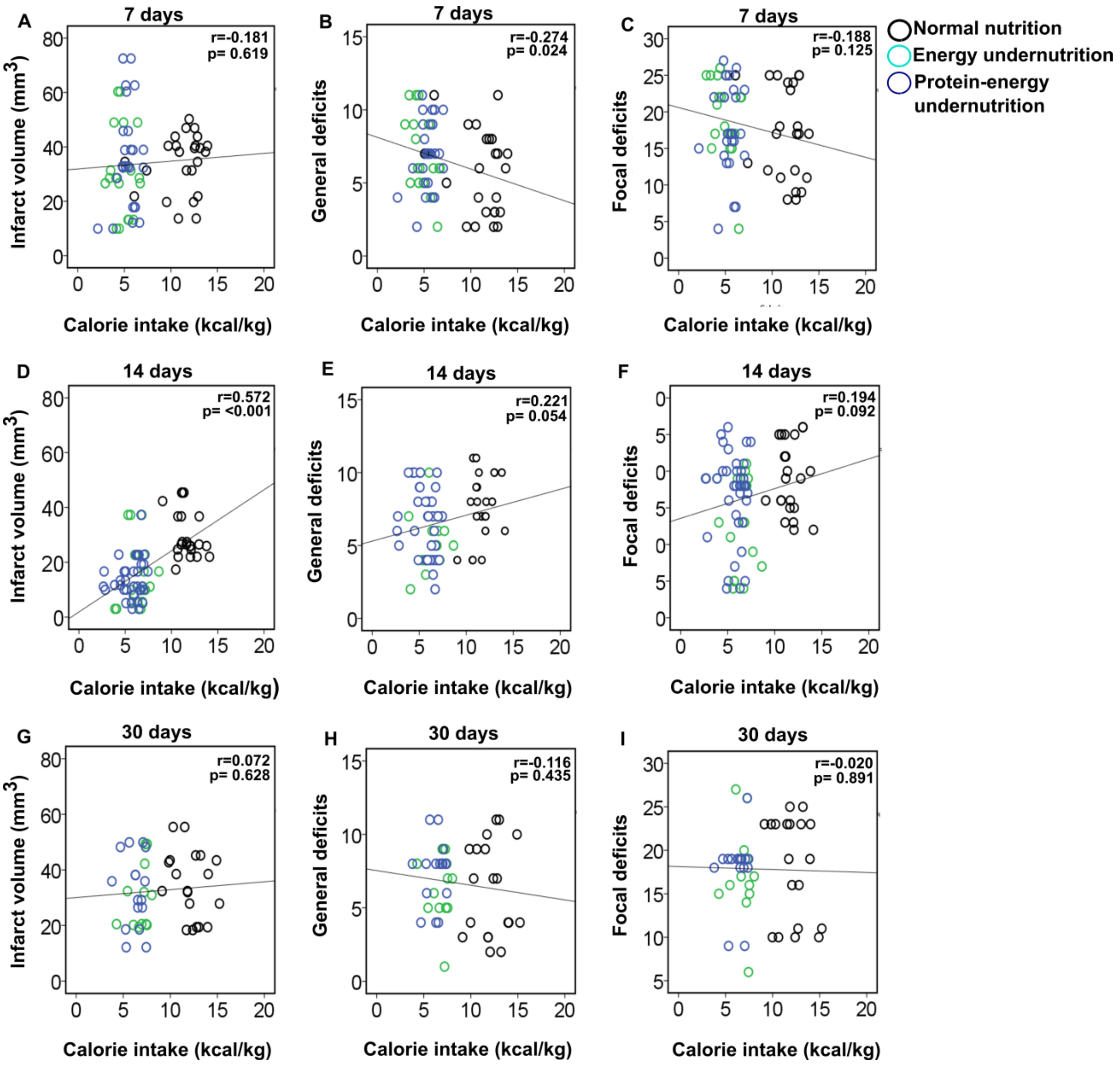

Supplemental Figure 3. Correlations of daily calorie intake with infarct volume and neurological deficits. Pearson's correlations of daily calorie intake with **(A, D, G)** infarct volume, **(B, E, H)** general neurological deficits and **(C, F, I)** focal neurological deficits in animals exposed to normal nutrition, energy **under**nutrition and protein-energy **under**nutrition for 7 days **(A-C)**, 14 days **(D-F)** or 30 days **(G-I)**, followed by 30 minutes intraluminal MCAO and 24 hours reperfusion. Correlation coefficients (r), p values and univariate regression lines are shown. Note the negative correlation of calorie intake with general neurological deficits 7 days after food manipulation and the positive correlation of calorie intake with infarct volume 14 days after food manipulation.

Supplemental Tables:

Supplemental Table 1. List of PCR primers

Primer		Sequence (5'->3')	Tm	G-C (%)	Gene bank number
Sirt-1	Forward	GATGACAGAACGTCACACGC	59.56	55.00	NM_019812.3
	Reverse	ATTGTTCGAGGATCGGTGCC	60.46	55.00	
Igf-1	Forward	GACTCAGAAGTCCCCGTCCC	61.61	65.00	NM_010512.5
	Reverse	GCATTTTCTGCTCCGTGGG	59.49	57.89	
Insr	Forward	ACCTTCTCTGATGAACGGCG	60.11	55.00	NC_000074.6
	Reverse	CTGATATGGGATCCAGGGGG	58.71	60.00	
Glut-1	Forward	GTTAATCGCTTTGGCAGGCGG	62.78	57.14	NM_011400.3
	Reverse	AGCATCTCAAAGGACTTGCCC	60.62	52.38	
Glut-2	Forward	GTGCTGCTGGATAAATTCGCC	60.27	52.38	NM_031197.2
	Reverse	TCAGCAACCATGAACCAAGGG	60.82	60.82	
Il-1β	Forward	TCTTTGAAGTTGACGGACCCC	60.20	52.38	NC_000068.7
	Reverse	CTTGTTGATGTGCTGCTGCG	60.73	55.00	
Nf-κb	Forward	TTTCGACTACGCAGTGACGG	60.39	55.00	NM_008689.2
	Reverse	GCTAAGTGTAAGACACTGTCCC	58.41	50.00	
Nox-4	Forward	CCTGCTCATTTGGCTGTCCC	59.96	50.00	NM_015760.5
	Reverse	GCTTAAACACAATCCTAGGCCC	59.97	55.00	
Sod-1	Forward	CATCCACTTCGAGCAGAAGGC	61.34	57.14	NM_011434.1
	Reverse	GGTACAGCCTTGTGTATTGTCCC	61.18	52.17	
Sod-2	Forward	GAACAACAGGCCTTATTCCGC	61.32	60.00	NM_013671.3
	Reverse	GTGTATCTTTCAGTAACATTCTCCC	59.31	50.00	
Gpx-3	Forward	GCACTACAAGAAGAACTTGGGC	59.77	50.00	NM_001329860.1
	Reverse	TCGAACATACTTGAGACTGGGG	59.50	50.00	
Cat	Forward	TGGTATAAGACGCATCAGAAGCC	60.49	47.83	NC_000071.6
	Reverse	GGTACTCCTCACTGAACATGCG	60.99	54.55	
β-Gluc	Forward	TGGTATAAGACGCATCAGAAGCC	60.49	47.83	NC_000071.6
	Reverse	GGTACTCCTCACTGAACATGCG	60.99	54.55	

Supplemental Table 2. General observations in mice exposed to energy and protein-energy undernutrition

Clinical observations	Stool size (mm)	Stool color loss (%)	Stool blood beddings (%)	Mild hypoactivity (%)
7 days				
Normal nutrition	3.9±1.1	0	0	0
Energy undernutrition	7.0±0.8***	100	0	7.1
Protein-energy undernutrition	6.6±0.9***	100	7.1	21.4
14 days				
Normal nutrition	2.9±0.3	0	0	0
Energy undernutrition	7.4±0.5***	100	5.6	22
Protein-energy undernutrition	8.9±2.0***	100	5.6	5.6
30 days				
Normal nutrition	3.4±0.6	0	0	0
Energy undernutrition	9.1±1.2***	100	15.4	38.5
Protein-energy undernutrition	14.3±1.3***	100	30.8	54

***p<0.001 compared with corresponding normal nutrition.

Supplemental Table 3. Plasma lipid and glucose levels in mice exposed to energy and protein-energy undernutrition

	Cholesterol (mg/dl)	LDL (mg/dl)	Triglycerides (mg/dl)	Glucose (mg/dl)
7 days				
Normal nutrition	244.0±94.0	30.4±20.0	216.0±142.0	89.4±51.0
Energy undernutrition	253.0±108.0	25.3±18.0	224.0±113.0	84.4±40.3
Protein-energy undernutrition	219.4±53.0	11.6±3.3*	197.0±69.0	77.1±25.7
14 days				
Normal nutrition	244.5±130.3	22.5±23.0	225.0±155.9	115.0±53.7
Energy undernutrition	208.0±99.1	20.0±21.5	244.0±126.1	97.5±52.0
Protein-energy undernutrition	270.0±110.1	25.5±22.5	256.0±107.7	100.0±27.7
30 days				
Normal nutrition	252.1±120.0	32.7±29.7	259.3±147.2	97.7±46.0
Energy undernutrition	317.8±101.0	30.5±17.3	333.3±113.7	73.6±50.0
Protein-energy undernutrition	218.6±94.8	20.7±13.0	198.6±130.4	85.0±23.5

*p<0.05 compared with corresponding normal nutrition.
